# Therapy-related myeloid neoplasms following chimeric antigen receptor T-cell therapy for Non-Hodgkin Lymphoma

**DOI:** 10.1038/s41408-022-00707-4

**Published:** 2022-07-26

**Authors:** Hassan B. Alkhateeb, Razan Mohty, Patricia Greipp, Radhika Bansal, Matthew Hathcock, Allison Rosenthal, Hemant Murthy, Mohamed Kharfan-Dabaja, Jose C. Bisneto Villasboas, Nora Bennani, Stephen M. Ansell, Mrinal M. Patnaik, Mark R. Litzow, Rong He, Dong Chen, Aref Al-Kali, Saad S. Kenderian, Yi Lin, Mithun Vinod Shah

**Affiliations:** 1 Division of Hematology, Mayo Clinic, Rochester, MN USA; 2 Department of Hematology/Oncology, Mayo Clinic, Jacksonville, FL USA; 3 Division of Hematopathology, Mayo Clinic, Rochester, MN USA; 4 Division of Hematology/Oncology, Mayo Clinic, Scottsdale, AZ USA

**Keywords:** Translational research, Haematological cancer

Dear Editor,

Chimeric antigen receptor T-cell therapy (CAR-T) is a novel class of therapeutics with expanding indications. Currently, CAR-T is FDA approved for relapsed, refractory B-cell acute lymphoblastic leukemia, various subtypes of non-Hodgkin lymphoma (NHL), and multiple myeloma [1].

Therapy-related myeloid neoplasms (t-MN) are aggressive leukemia and associated with high-risk features including complex karyotype (CK), monosomal karyotype (MK), and pathogenic variants (PV) in *TP53*. Consequentially, the survival following t-MN is generally <1 year. Traditionally, the development of t-MN is associated with exposure to DNA-damaging agents, such as alkylators and radiation, and develops at an average of 5–7 years following the exposure [2, 3]. Recently, exposure to novel modalities such as poly-ADP ribose polymerase (PARP)-inhibitors [4] and peptide receptor radionuclide therapy (PRRT) [5] have been linked to t-MN development.

NHL is one of the most common primary malignancies associated with the risk of a subsequent t-MN. In a series of NHL patients who underwent autologous stem cell transplant (SCT), the 10-year incidence of t-MN was 6% [6]. Older age, having received more lines of therapy, and the use of total body irradiation as a part of the conditioning regimen was associated with a higher risk of t-MN.

The pivotal CAR-T studies in NHL did not report the development of second primary malignancies, including t-MN [7–9]. In contrast, recent reports suggested t-MN developing at a median of 3–6 months. However, a systematic study of t-MN following CAR-T and the outcomes thereafter has not been performed. Here, we present the characteristics and outcomes of patients who developed t-MN following CAR-T for NHL and compare outcomes to patients who developed t-MN post SCT.

Detailed methods are provided in Supplementary Methods. Between January 1, 2018, and December 31, 2021, 189 patients underwent commercially available CAR-T (93% axicabtagene ciloleucel, 4% brexucabtagene autoleucel, 2% tisagenlecleucel, 1% lisocabtagene maraleucel) for relapsed/refractory NHL at Mayo Clinic. Of these, 10 (5.3%) developed t-MN—five (50%) of whom had received prior autologous SCT, whereas the rest (50%) did not. In those receiving both SCT and CAR-T, the median interval from SCT to CAR-T was 9.8 months (IQR 8.7–23.1). Median interval from CAR-T day 0 to t-MN diagnosis was 9.1 months (IQR 3.6–19.8), with 6 (60%) patients developing t-MN within 1 year from CAR-T. Among the 120 patients with complete follow-up available, the cumulative incidences (CI) of t-MN at 1 and 2 years were 5% and 11%, respectively (Fig. 1A).

**Fig. 1:**
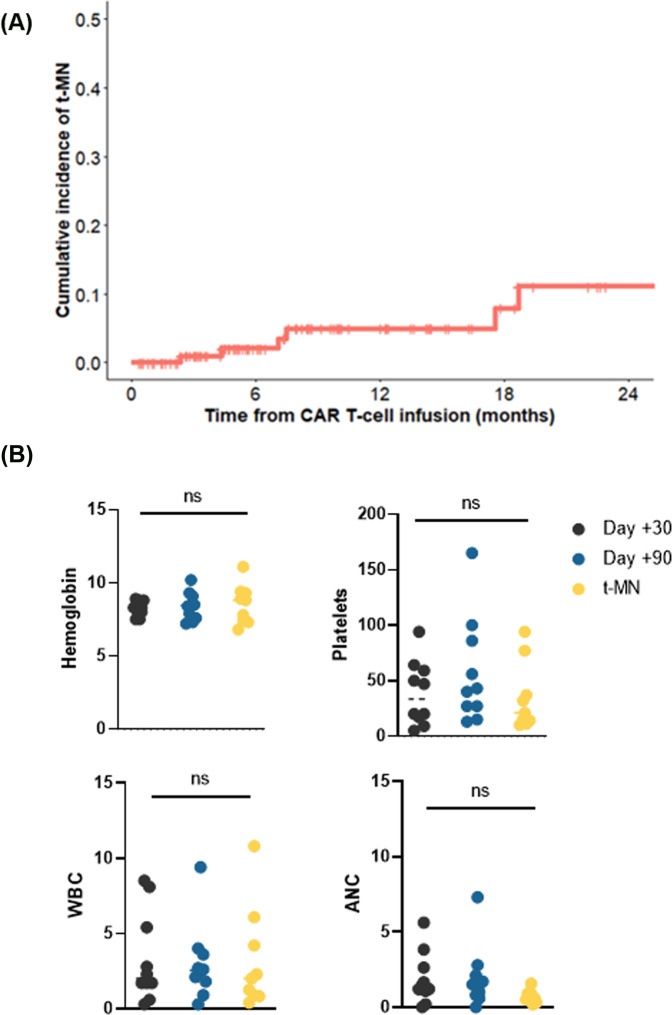
Cumulative incidence and cytopenia associated with therapy-related myeloid neoplasm (t-MN) development in non-Hodgkin Lymphoma patients undergoing chimeric antigen receptor (CAR)-T therapy. **A** Cumulative incidence of t-MN in NHL patients undergoing CAR T cell therapy at 2 years. **B** Complete blood count at day +30 post CAR T-cell therapy, day +100 post-CAR T-cell therapy, and at t-MN diagnosis. Hemoglobin in g/dL, platelets ×10^9^/L, white blood cell count (WBC)—×10^9^/L, and absolute neutrophil count (ANC)—×10^9^/L.

Clinical and laboratory characteristics of patients who developed t-MN following CAR-T are described in Supplementary Table 1. Prior to CAR-T, all patients underwent bone marrow aspirate and biopsy evaluation. At the time of pre-CAR-T bone marrow evaluation, median hemoglobin was 9.9 g/dL (IQR 8.8–10.7), white blood cell (WBC) count was 3.5 × 10^9^/L (IQR 2.5–4.3), absolute neutrophil count (ANC) was 2.2 × 10^9^/L (IQR 1.3–2.9), and platelet count was 115 × 10^9^/L (IQR 61–168). Upon morphology review of pre-CAR-T bone marrow, none were noted to have morphologically diagnostic features of t-MN. We retrospectively performed NGS using the pre-CAR-T bone marrow sample in three patients. All three patients harbored PVs: patient #1251 had *DNMT3A* (Arg882His, VAF 7%), patient# 1724 had *DNMT3A* (Met801Val, VAF 8%), and patient# 2035 had *TP53* (Ile254Ser, VAF 40%).

All patients received fludarabine and cyclophosphamide lymphodepletion chemotherapy followed by the infusion of axicabtagene ciloleucel. Peak cytokine release syndrome (CRS), neurotoxicity, and response rates at day +30 and +90 are provided in Supplementary Table 1. Median hemoglobin, WBC, ANC, and platelet counts at day +30 were 8.3 (IQR 8–8.7), 2 (IQR 1.7–4.8), 1.3 (IQR 1.1–2.4), 33.5 (17.8–56.8) and at day +100 was 8.55 (IQR 7.7–9), 2.55 (IQR 1.9–3.4), 1.5 (IQR 0.9–2), and 41.5 (IQR 27–78.5), respectively (Fig. 1B).

At t-MN diagnosis, the median age was 66.2 years and 70% were female (Supplementary Table 2). Eight (80%) patients presented with t-MDS, whereas 20% presented as t-AML. CBC parameters at t-MN diagnosis were not different from day +30/+90 assessments: hemoglobin 8.8 (IQR 7.4–9.3), WBC was 2 (IQR 0.9–4.2), ANC was 0.7 (IQR 0.5–1), and platelet count was 21 (IQR 14–37). Nine (90%) patients had a cytogenetic abnormality, of which 4 (40%) had CK/MK. NGS at t-MN diagnosis was available in 9 (90%) patients with *TP53* being the most commonly mutated gene (4, 44.4%). Paired genetic analysis of pre-CAR-T and t-MN bone marrows (*n* = 3) showed that *DNMT3A* clones detected in the pre-CAR-T bone marrow were not detectable at t-MN in both patients—one (UPIN# 1251) developed *RUNX1*-mutated t-MN with deletion 20q, and the other (UPIN# 1724) had deletion 13q, but no detectable PVs. UPIN# 2035 was noted to have the expansion of the *TP53* clone (VAF 40 to 84%) and CK/MK at t-MN diagnosis.

We next compared t-MN that developed following the two cellular therapy platforms: patients who developed t-MN following SCT (SCT t-MN cohort, *n* = 28) and CAR T-cell therapy (CAR-T t-MN cohort, *n* = 10). As noted above, 5 of the 10 CAR-T t-MN patients had received a prior autologous SCT. The clinical and laboratory characteristics of the two cohorts are shown (Supplementary Table 3). CAR-T t-MN cohort patients received more lines of therapy (5 vs. 4, *P* = 0.02), though the cumulative doses of doxorubicin (262.5 *vs*. 300 mg/m^2^, *P* = 0.025) and melphalan (70 vs. 140 mg/m^2^, *P* = 0.01) were lower. The cumulative doses of the other alkylators as well as the proportion of the patients who received platinum-based or nucleoside analog-based chemotherapies were not different. At t-MN diagnosis, CAR-T t-MN patients had lower hemoglobin (6.8 vs. 8.7 g/dL, *P* = 0.008), ANC (2 vs. 4.6 × 10^9^/L, *P* = 0.016, and platelet count (21 vs. 65 × 10^9^/L, *P* = 0.009). Cytogenetic and genetic profiles including the proportions of patients with CK, MK, and PV in *TP53* were not different between the two cohorts.

The median myeloid-neoplasm-free survival (MNFS) from the first intervention was shorter for CAR-T t-MN patients compared to SCT t-MN patients (43 vs. 100 months, *P* = 0.01, Fig. 2A). This observation remained valid when patients who received both SCT and CAR-T (*n* = 5) were excluded (29 vs. 100 months, *P* = 0.01, Fig. 2B). Similarly, the median MNFS from day 0 for CAR-T t-MN cohort was shorter compared to the SCT t-MN cohort—both when patients who received both SCT and CAR-T were included (22 vs. 44 months, *P* = 0.01, Fig. 2C), or excluded (11 vs. 44 months, *P* < 0.001, Fig. 2D). The median MNFS for CAR-T t-MN was not different when stratified by having received prior SCT (*n* = 5) or not (*n* = 5, 27 vs. 11 months, *P* = 0.11), though this analysis was limited by small sample size. Finally, overall survival following t-MN development was not different between the cohorts (Fig. 2E, F).

**Fig. 2:**
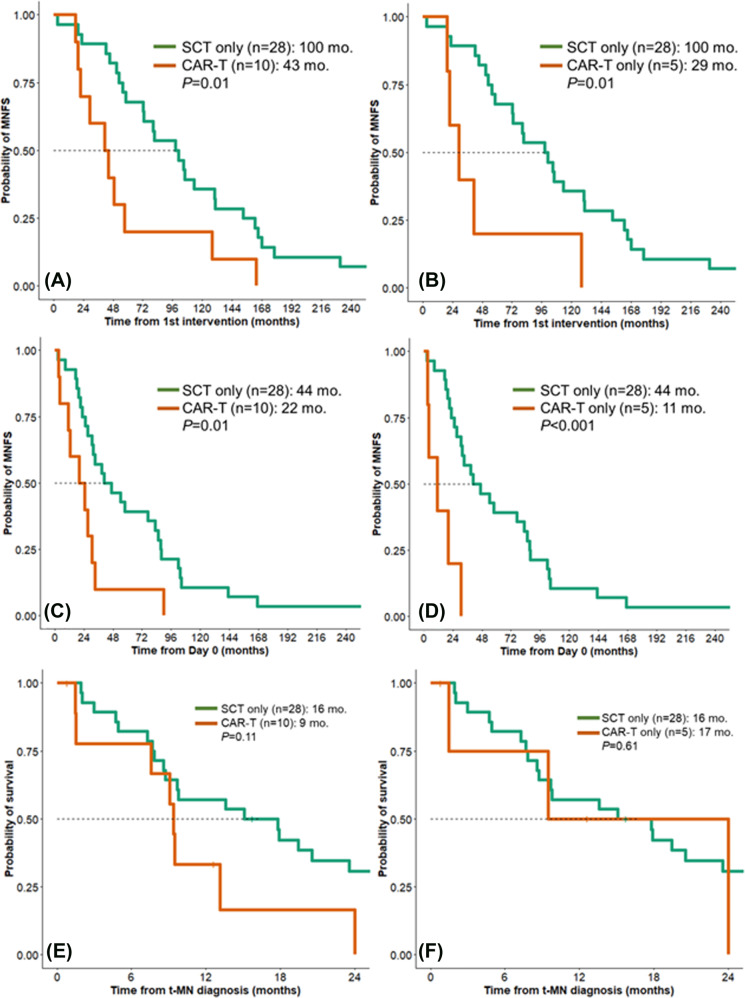
The latency to develop therapy-related myeloid neoplasm is significantly shorter following chimeric antigen receptor (CAR)-T therapy compared to autologous stem cell transplant (SCT) in NHL. **A** Myeloid neoplasm-free survival (MNFS) from the first intervention comparing patients who underwent SCT (*n* = 28) with those who underwent CAR T cell therapy with or without prior SCT (CAR-T, *n* = 10). **B** MNFS from the first intervention comparing patients who underwent SCT only (*n* = 28) or CAR-T only (*n* = 5). **C** MNFS from day 0 comparing patients who underwent SCT (n = 28) with those who underwent CAR-T with or without prior SCT (*n* = 10). **D** MNFS from day 0 comparing patients who underwent SCT only (*n* = 28) or CAR-T only (*n* = 5). **E** Comparing OS from t-MN diagnosis in patients who underwent SCT (*n* = 28) with those who underwent CAR-T with or without prior SCT (*n* = 10), and **F** comparing OS from t-MN diagnosis in patients who underwent SCT only (*n* = 28) or CAR-T only (*n* = 5).

Our two most notable observations include a relatively high incidence of t-MN and the short interval from CAR-T to t-MN. No prior studies have reported the CI of t-MN following CAR-T. On the other hand, short latency appears to be a common theme in post-CAR-T t-MN. For example, Shouse et al. identified four patients who developed t-MN at a median interval of 3 months (range 2–3) [10], and Cordeiro et al. observed 4 of 86 patients developed t-MN at a median of 6 months (range 4–17) [11]. In both cases, a subset of patients was noted to have evidence of dysplasia [10] or clonal abnormalities prior to CAR-T therapy [11].

Potential explanations for the shorter latency and a substantially higher CI of t-MN following CAR-T compared to historical observations therapies [3, 12, 13], include the additional DNA-damaging therapy received, improved survival due to better lymphoma control, or that CAR-T, by yet an unknown mechanism, increased the risk of t-MN. While patients who received CAR-T indeed received a median of one additional line of therapy, cumulative exposures to the known DNA-damaging agent were not different. Second, the interval from day 0 to t-MN was not different between CAR T-cell patients whether they received prior SCT or not. Finally, we consistently observed shorter MNFS in the CAR-T t-MN cohort—both when calculated from the first intervention or day 0. However, proving the causality of t-MN is challenging [12], especially as NHL patients receive multiple lines of DNA-damaging therapies over a variable interval. Moreover, there is a debate that SCT acts as an additional risk factor for t-MN [6, 12]. Given that half of the CAR T-cell t-MN cohort also received prior SCT, this small retrospective analysis does not allow for establishing CAR-T as a contributory factor for t-MN and the mechanism underlying our observations remains undetermined. Other limitations of our study include those of single-institution retrospective studies. Second, it has not been our practice to obtain cytogenetics and NGS on all NHL patients undergoing CAR-T, limiting our ability to track the clonal evolution before and after CAR-T.

Our observations have notable implications. First, given the strikingly short interval between CAR-T and the development of t-MN, all patients undergoing CAR-T for NHL should have a careful discussion regarding this potentially fatal complication. Second, we and others noted that a subset of patients had dysplasia [10] or clonal abnormalities [11, 14] before CAR-T. Therefore, a careful bone marrow evaluation including cytogenetics and NGS may be beneficial [14]. Finally, as the utilization of CAR-T increases; surveillance, diagnosis, and treatment of t-MN should be an essential component of the long-term survivorship plan.

## Supplementary information

Supplementary Material

Author checklist
