# Supplementary material for: Therapy-related myeloid neoplasms following chimeric antigen receptor T-cell therapy for Non-Hodgkin Lymphoma
Source: Blood Cancer J. 2022 Jul 26;12(7):113. doi: 10.1038/s41408-022-00707-4 (PMC9325766; doi:10.1038/s41408-022-00707-4)
Supplement: Supplementary file 1 — Supplementary Material [file 41408_2022_707_MOESM1_ESM.pdf]

1. Supplementary Methods

The study was approved by the Institutional Review Board of Mayo Clinic. After obtaining informed consent, we retrospectively identified all patients with histologically confirmed large B-cell lymphoma including diffuse large B-cell lymphoma (DLBCL), primary mediastinal B-cell lymphoma, or transformed follicular lymphoma (FL) between January 1, 2018 to December 31, 2021, using the commercially available chimeric antigen receptor (CAR) T-cell therapy and later developed World Health Organization-defined therapy-related myeloid neoplasm (t-MN) (1). We also identified all NHL patients who underwent stem cell transplantation (SCT) and developed t-MN. Lines of therapy and cumulative doses of the known DNA-damaging agents were calculated from NHL diagnosis to the t-MN development, including both SCT and CAR-T as a line of therapy as applicable.

All patients underwent a bone marrow evaluation prior to CAR T-cell infusion as well as at the time of t-MN diagnosis. All available pre-CAR-T bone marrow aspirate smears and biopsies were re-reviewed by a hematopathologist (D.C.) to exclude the presence of t-MN. The work up at t-MN diagnosis included cytogenetic and the next-generation sequencing (NGS) analysis using 42 genes commonly mutated in myeloid neoplasms: *ANKRD26, ASXL1, BCOR, CALR, CBL, CEBPA, CSF3R, DDX41, DNMT3A, ELANE, ETNK1, ETV6, EZH2, FLT3, GATA1, GATA2, IDH1, IDH2, JAK2, KDM6A, KIT, KRAS, MPL, NPM1, NRAS, PHF6, PTPN11, RAD21, RUNX1, SETBP1, SH2B3, SF3B1, SRP72, SMC3, SRSF2, STAG2,TERT, TET2, TP53, U2AF1, WT1*, and *ZRSR2*. The library preparation, sequencing and data analysis were performed as described.(2) Briefly, libraries were prepared using the Agilent SureSelect-XT Target Enrichment Kit (SureSelectXT, Agilent, Santa Clara, CA). and sequencing was performed on MiSeq or HiSeq platforms (Illumina, San Diego, CA). Pathogenic and likely pathogenic variants calling was performed as described. The limit of detection of the NGS assay was variance allele frequency

(VAF) 5% with a minimum 250x coverage. More than 95% of the tested regions had >1000x coverage in the clinical assay (2, 3).

Cytokine release syndrome and neurotoxicity were assessed using the American Society for Transplant and Cellular Therapy Consensus Grading for Cytokine Release Syndrome and Neurologic Toxicity Associated with Immune Effector Cells criteria (4). Responses at day +30 and +90 following CAR-T were assessed using the International Working Group Response Criteria for Malignant Lymphoma (5).

Continuous and categorical variable were compared using the Kruskal-Wallis and Fisher Exact tests, respectively. The cumulative incidence of t-MN was calculated from CAR T-cell infusion to the t-MN development with death from other causes as a competing risk. Myeloid neoplasm-free survival was calculated from the time of the first intervention (date of the first treatment for NHL) or from day 0 (for SCT or CAR-T as applicable) to the development of t-MN as applicable. In the subset of patients who underwent SCT followed by CAR-T, the first day 0 (day 0 for SCT) was used for survival analysis. Overall survival was estimated from t-MN diagnosis to death or last follow up, whichever came first using the Kaplan-Meier method. Statistical analysis was performed using BlueSky Software (version 7.4, Chicago, USA).

REFERENCES

1. Arber DA, Orazi A, Hasserjian R, Thiele J, Borowitz MJ, Le Beau MM, et al. The 2016 revision to the World Health Organization classification of myeloid neoplasms and acute leukemia. Blood. 2016;127(20):2391-405.
2. He R, Devine DJ, Tu ZJ, Mai M, Chen D, Nguyen PL, et al. Hybridization capture-based next generation sequencing reliably detects FLT3 mutations and classifies FLT3-internal tandem duplication allelic ratio in acute myeloid leukemia: a comparative study to standard fragment analysis. Modern Pathology. 2020;33(3):334-43.
3. Mehta N, He R, Viswanatha DS. Internal Standardization of the Interpretation and Reporting of Sequence Variants in Hematologic Neoplasms. Molecular Diagnosis & Therapy. 2021;25(4):517-26.
4. Lee DW, Santomasso BD, Locke FL, Ghobadi A, Turtle CJ, Brudno JN, et al. ASTCT Consensus Grading for Cytokine Release Syndrome and Neurologic Toxicity Associated with Immune Effector Cells. Biology of Blood and Marrow Transplantation. 2019;25(4):625-38.
5. Cheson BD, Pfistner B, Juweid ME, Gascoyne RD, Specht L, Horning SJ, et al. Revised Response Criteria for Malignant Lymphoma. Journal of Clinical Oncology. 2007;25(5):579-86.

2. Supplementary Tables

Supplementary Table 1: Clinical and laboratory characteristics of non-Hodgkin lymphoma patients who developed therapy-related myeloid neoplasm following chimeric antigen receptor T-cell therapy

Supplementary Table 2: Characteristics and outcomes therapy-related myeloid neoplasms following chimeric antigen receptor T-cell therapy

Supplementary Table 3: Clinical and laboratory characteristics of non-Hodgkin lymphoma patients that developed therapy-related myeloid neoplasm following stem cell transplant or chimeric antigen receptor T-cell therapy

Supplementary Table 1: Clinical and laboratory characteristics of non-Hodgkin lymphoma patients who developed therapy-related myeloid neoplasm following chimeric antigen receptor T-cell therapy

Patient ID#	1057	1147	1251	1724	1725	2006	2008	2035	2056	2057
Primary malignancy(ies)	DLBCL	DLBCL, endometrial carcinoma	DLBCL	Transformed FL	High-grade lymphoma	DLBCL	DLBCL	Transformed FL, breast cancer	DLBCL	DLBCL
Age at lymphoma diagnosis (years)	60.1	52.2	65.3	44.8	67.4	55.0	64.4	59.4	63.9	59.4
Gender	Male	Female	Female	Female	Female	Female	Male	Female	Male	Female
Lines of therapy received	5	5	5	7	5	5	8	4	10	4
Prior SCT	Yes	Yes	Yes	No	No	No	Yes	No	Yes	No
Interval from SCT to CAR T-cell therapy (months)	8.7	82.8	6.4	NA	NA	NA	23.1	NA	9.8	NA
Interval from CAR-T cell therapy to t-MN (Months)	18.7	7	2.4	30.6	3.3	4.3	1.7	20.2	23.6	11.2
**Bone marrow evaluation prior to CAR-T cell therapy**										
Hemoglobin (g/dL)	9.50	8.50	8.20	7.70	10.4	10.8	13.3	9.8	10.9	9.6
MCV	97.6	88.1	88.7	88.5	106.1	91.8	99.0	99.1	97.0	123.4
RDW	13.4	18.0	14.0	18.1	13.7	13.3	14.6	14.8	13.2	13.7
WBC ($x10^9$/L)	2.4	1.8	2.7	1.1	4.4	3.5	4.4	3.9	6.5	3.4
ANC ($x10^9$/L)	1.58	1.19	2.67	0.02	0.90	2.98	3.08	2.81	4.16	1.76
Platelet count ($x10^9$/L)	94	326	36	4	171	106	123	286	159	51
ALC ($x10^9$/L)	0.336	0.288	0.7	0.253	0.109	0.035	0.748	0.273	1.69	1.93
AMC ($x10^9$/L)	0.456	0.234	1.3	0.004	0.087	0.385	0.44	0.351	0.455	0.152
Bone marrow blasts %	0	0	0	2.6	3.5	0	0	0	0	0
Bone marrow cellularity (%)	30	40	70	70	30	20	30	40	60	30
Myeloid: erythroid ratio	NA	3:1	4:1	11:1	0.8:1	NA	3:1	Normal	2.2:1	1:1
Erythroid precursor quantity	Increased	Normal	Normal	Decreased	Normal	Decreased	Normal	Normal	Normal	Normal
Erythroid dysplasia	Absent	Absent	Absent	Absent	Absent	Absent	Absent	Absent	Absent	Absent
Myeloid precursor quantity	Moderately decreased	Normal with left shift	Normal	Decreased	Normal	Decreased	Normal	Normal	Normal	Normal
Myeloid dysplasia	Absent	Absent	Absent	Absent	Absent	Absent	Absent	Absent	Absent	Absent
Megakaryocytic precursor quantity	Normal	Normal	Normal	Decreased	Normal	Normal	Normal	Normal	Normal	Normal
Megakaryocytic dysplasia	Absent	Absent	Absent	Absent	Absent	Absent	Absent	Absent	Absent	Absent
NGS	ND	ND	*DNMT3A* (7%)	*DNMT3A* (8%)	ND	ND	ND	*TP53* (40%)	ND	ND

Percentage bone marrow involvement by lymphoma	0	0	40	80	Low level cannot be ruled out	0	0	<10	60	0
**Characteristics of CAR-T therapy**										
CAR-T cell product	axicabtagene	axicabtagene	axicabtagene	axicabtagene	axicabtagene	axicabtagene	axicabtagene	axicabtagene	axicabtagene	axicabtagene
Lymphodepletion therapy	Flu/Cy	Flu/Cy	Flu/Cy	Flu/Cy	Flu/Cy	Flu/Cy	Flu/Cy	Flu/Cy	Flu/Cy	Flu/Cy
Peak cytokine release syndrome grade	1	1	1	2	1	0	0	1	1	2
Peak neurotoxicity grade	0	1	0	4	0	0	0	2	0	3
Day +30 response	CR	Prog.	PR	PR	PR	Prog.	PR	CR	PR	CR
Day +30 hemoglobin (g/dL)	7.5	8.8	8.8	7.5	8	8.4	8	8.3	8.9	8.3
Day +30 WBC (x10^9/L)	1.7	5.4	2.3	1.7	2.8	1.7	0.6	0.3	8.5	8.1
Day +30 ANC (x10^9/L)	1.2	3.834	1.66	1.5	1.2	1.1	0.2	0	5.6	2.6
Day +30 platelet count (x10^9/L)	20	59	20	9	94	64	50	5	47	17
Day +100 response	CR	Prog.	PR	CR	PR	Prog.	Prog.	CR	Prog.	CR
Day +90 hemoglobin (g/dL)	7.9	7.6	7.3	9.3	7.2	10.2	8.4	8.5	9.1	8.5
Day +90 WBC (x10^9/L)	3.6	9.4	0.9	1.8	2.7	2.6	0.3	2.1	4	2.5
Day +90 ANC (x10^9/L)	2.8	7.3	0.6	1	1.5	1.7	0	1.5	2.1	0.8
Day +90 platelet count (x10^9/L)	43	40	15	27	86	100	13	165	56	27

DLBCL – diffuse large B-cell lymphoma; FL – Follicular lymphoma; SCT – stem cell transplant; Flu – fludarabine; Cy – cyclophosphamide; CAR T-cell – chimeric antigen receptor T-cell therapy; MCV – mean corpuscular volume; RDW – red cell distribution width; WBC – white blood cell count; ANC – absolute neutrophil count; ALC – absolute lympohcoyte count; AMC – absolute monocyte count; CR – complete remission; PR – partial remission; prog. – progression; t-MN – therapy-related myeloid neoplasm; NGS – next-generation sequencing; ND – not done.

Supplementary Table 2: Characteristics and outcomes therapy-related myeloid neoplasms following chimeric antigen receptor T-cell therapy

Patient ID#	1057	1147	1251	1724	1725	2006	2008	2035	2056	2057
Age at t-MN diagnosis (years)	63.9	65.7	66.7	55.3	69.3	56.6	68.4	76.6	69.5	61.8
Phenotype	t-MDS	t-AML	t-MDS	t-MDS	t-MDS	t-MDS	t-AML	t-MDS	t-MDS	t-MDS
Hemoglobin (g/dL)	7.4	8.8	7.3	11.1	8.90	9.4	6.8	9.3	7.80	NA
WBC (x10^9/L)	0.83	10.80	0.9	1.26	6.08	2.30	0.40	2.00	4.20	NA
ANC (x10^9/L)	0.18	0.90	0.50	30.24	0.55	1.56		0.88	0.59	NA
Platelet count (x10^9/L)	21	32	15	37	77	94	11	10	14	NA
Bone marrow blasts (%)	2	24	4	1	5.4	18	83		0	NA
Cytogenetics	CK/MK	CK/MK	del20q	del13q	CK/MK	t(3;8), del7q	Monosomy 7	CK/MK	Del 7q, +21	Normal
NGS	*TP53* (49.3%)	ND	*RUNX1* (60%)	None	*TP53* (10%)	None	*IDH1* (17%)	*TP53* (84%)	None	*TP53* (6%), *TP53* (8%)
Vital status at last follow-up	Dead	Dead	Dead	Alive	Dead	Dead	Dead	Dead	Dead	Alive
Interval from t-MN to last follow up (months)	13.1	9.1	7.6	12.7	9.5	24.0	1.5	1.5	9.4	0.8
Primary cause of death	t-MN	t-MN	t-MN		t-MN	Infection	t-MN	t-MN	t-MN	

t-MN – therapy-related myeloid neoplasm; t-MDS – therapy-related myelodysplastic syndrome; t-AML – therapy-related acute myeloid leukemia; WBC – white blood cell count; ANC – absolute neutrophil count; CK – complex karyotype; MK – monosomal karyotype; NGS – next-generation sequencing; ND – not done.

Supplementary Table 3: Clinical and laboratory characteristics of non-Hodgkin lymphoma patients that developed therapy-related myeloid neoplasm following stem cell transplant or chimeric antigen receptor T-cell therapy

Variables	No prior CAR-T (n=28)	Prior CAR-T (N=10)	Total (N=38)	*P*-value
Age (years) at lymphoma diagnosis, median (Q1, Q3)	52.0 (43.4, 59.5)	59.8 (56.1, 64.3)	54.0 (46.1, 64.0)	0.091
Age (years) at t-MN diagnosis, median (Q1, Q3)	64.1 (53.2, 69.5)	66.2 (62.3, 69.1)	64.5 (55.6, 69.4)	0.407
No. of lines of therapy, median (Q1, Q3)	4.0 (3.0, 5.0)	5.0 (5.0, 6.5)	4.0 (3.0, 5.0)	0.022
Prior chemotherapy				
No	0 (0%)	0 (0%)	0 (0%)	1
Yes	28 (100.0%)	10 (100.0%)	38 (100.0%)	
Prior radiation				0.449
No	19 (67.9%)	5 (50.0%)	24 (63.2%)	
Yes	9 (32.1%)	5 (50.0%)	14 (36.8%)	
Prior autologous SCT				< 0.001
No	0 (0.0%)	5 (50.0%)	5 (13.2%)	
Yes	28 (100.0%)	5 (50.0%)	33 (86.8%)	
Cumulative cyclophosphamide dose (g/m^2)	6.0 (1.5, 6.0)	6.0 (6.0, 7.8)	6.0 (3.0, 7.0)	0.18
Cumulative iphosphamide dose (g/m2)	10 (0.0, 10.0)	5.0 (1.25, 10.0)	10.0 (0.0, 10.0)	0.789
Cumulative etoposide dose (mg/m2)	895.0 (400.0, 1060.0)	800.0 (400.0, 1450.0)	895.0 (400.0, 1285.0)	0.798
Cumulative doxorubicin dose (mg/m2)	300.0 (300.0, 300.0)	262.5 (250.0, 300.0)	300.0 (250.0, 300.0)	0.025
Cumulative melphalan dose (mg/m2)	140.0 (140.0, 140.0)	70.0 (0.0, 140.0)	140.0 (140.0, 140.0)	0.01
Platinum-based chemotherapy				0.404
No	7 (25.9%)	1 (10.0%)	8 (21.6%)	
Yes	20 (74.1%)	9 (90.0%)	29 (78.4%)	
Missing (n)	1	0	1	
Nucleoside analogue				0.462
No	1 (3.6%)	1 (10.0%)	2 (5.3%)	
Yes	27 (96.4%)	9 (90.0%)	36 (94.7%)	
Phenotype of t-MN at diagnosis				0.271
acute myeloid leukemia	8 (28.6%)	2 (20%)	10 (26.3%)	
chronic myelomonocytic leukemia	1 (3.6%)	0	1 (2.6%)	
myelodysplastic syndrome	19 (67.9%)	8 (80%)	27 (71.1%)	
Hemoglobin at t-MN (g/dL), median (Q1, Q3)	8.7 (7.8, 11. 5)	6.8 (8.9, 8.8)	8.5 (7.4, 10.3)	0.008
WBC count at t-MN (x10^9/L), median (Q1, Q3)	4.6 (2.5, 9.8)	2.0 (0.9, 4.2)	4.2 (2.2, 8.9)	0.016
Absolute neutrophil count at t-MN (x10^9/L), median (Q1, Q3)	1.8 (0.9, 2.9)	0.7 (0.5, 1.1)	1.5 (0.6, 2.9)	0.136
Platelet count at t-MN, median (x10^9/L) (Q1, Q3)	65.0 (31.5, 181.0)	21.0 (14.0, 37.0)	46.0 (26.0, 96.0)	0.009
Percent bone marrow blasts at t-MN, median (Q1, Q3)	5.5 (2.0, 15.6)	4.7 (1.8, 19.5)	5.2 (2.0, 17.6)	0.985
Chromosome 5 abnormality				0.469
No	15 (53.6%)	7 (70.0%)	22 (57.9%)	
Yes	13 (46.4%)	3 (30.0%)	16 (42.1%)	
Chromosome 7 abnormality				1
No	12 (42.9%)	4 (40.0%)	16 (42.1%)	
Yes	16 (57.1%)	6 (60.0%)	22 (57.9%)	
Chromosome 17 abnormality				0.168
No	22 (78.6%)	10 (100.0%)	32 (84.2%)	
Yes	6 (21.4%)	0 (0.0%)	6 (15.8%)	
Complex karyotype				0.468
No	12 (42.9%)	6 (60.0%)	18 (47.4%)	
Yes	16 (57.1%)	4 (40.0%)	20 (52.6%)	
PV in *FLT3*				1
Missing (n)	7	1	8	
No	20 (95.2%)	9 (100.0%)	29 (96.7%)	
Yes	1 (4.8%)	0 (0.0%)	1 (3.3%)	
NGS performed				
Missing (n)	9	1	10	
Yes	19	9	28	
TP53 PV*	8 (42.1%)	4 (44.4%)	12 (42.9%)	1

TET2 PV*	1 (5.3%)	0 (0.0%)	1 (3.6%)	1
ASXL1 PV*	2 (10.5%)	0 (0.0%)	2 (7.1%)	1
DNMT3A PV*	2 (10.5%)	0 (0.0%)	2 (7.1%)	1
RAS PV*	3 (15.8%)	0 (0.0%)	3 (10.7%)	0.53
RUNX1 PV*	1 (5.3%)	1 (11.1%)	2 (7.1%)	1

*Percentage of patients with available NGS.

t-MN – therapy-related myeloid neoplasm; SCT – stem cell transplant; CAR-T – chimeric antigen receptor (CAR) T-cell therapy; NGS – next-generation sequencing; PV – pathogenic variant
